# Racial and Ethnic Disparities in the Monetary Value of Informal Caregiving for Non-Institutionalized People Living With Dementia

**DOI:** 10.1177/08982643241262917

**Published:** 2024-06-17

**Authors:** Phillip Cantu, Tsai-Chin Cho, Mary Wyman, Brooke Helppie-McFall, Kristine J. Ajrouch

**Affiliations:** 1 Department of Internal Medicine-Geriatrics, 12338 University of Texas Medical Branch, Galveston, TX, USA; 2 Department of Internal Medicine, School of Medicine, 1259 University of Michigan, Ann Arbor, MI, USA; 3 Department of Psychology, School of Medicine and Public Health, 5228 University of Wisconsin, Madison, Madison, WI, USA; 4 Survey Research Center 1259 , Institute for Social Research, University of Michigan, Ann Arbor, MI, USA; 5 Department of Sociology, Anthropology, and Criminology, 8759 Eastern Michigan University, Ypsilanti, MI, USA

**Keywords:** caregiving, dementia, healthcare costs

## Abstract

**Objective:**

To examine racial and ethnic differences in costs of informal caregiving among older adults with dementia in the United States.

**Methods:**

We used data from the 2002 to 2018 Health and Retirement Survey to estimate annual informal care hours for adults with dementia (*n* = 10,015). We used regression models to examine racial and ethnic differences in hours of informal care for activities of daily living (ADL) and instrumental ADL, controlling for demographic characteristics, education, and level of disability.

**Results:**

Our sample was 70% non-Hispanic White, 19% non-Hispanic Black, and 11% Hispanic. Hispanics received, on average, 35.8 hours of informal care each week, compared to 30.1 for Blacks and 20.1 for Whites. Racial and ethnic differences persisted when controlling for covariates.

**Discussion:**

Informal care is a greater cost to racial and ethnic minoritized families. Informal care was valued at a replacement cost of $44,656 for Hispanics, $37,508 for Blacks, and $25,121 for Whites.

## Introduction

Because of significant and debilitating symptoms as well as a slow, progressive, extended course, Alzheimer’s disease and related dementias (ADRDs) have a profound impact on individuals, families, and society. While acute events, such as stroke, can result in rapid onset severe dementia, the majority of adults who experience dementia have extended periods of decline (Kelley et al., 2009; Rost et al., 2022). An estimated 6.5 million people over age 65 are currently living with Alzheimer’s disease in the United States (Alzheimer’s Association, 2022), and dementia prevalence is higher in racial and ethnic minoritized groups than in the non-Hispanic White (henceforth White) population (Manly et al., 2022; Mayeda et al., 2016; Rajan et al., 2021; Zhang et al., 2016).

Most care for people living with dementia (PwD) is provided by unpaid family and friends (*informal caregiving*) (Friedman et al., 2015). Work performed by informal caregivers occurs mostly without compensation, but it also carries a cost in foregone wages, opportunity for education or career advancement, and added psychosocial stress (Choi et al., 2021; Hurd et al., 2013; Langa et al., 2001). Individual caregivers do not bear these costs alone; the financial security and well-being of their larger family networks are also affected (Brodaty & Donkin, 2009; Lindeza et al., 2020).

The monetary value of unpaid *informal* caregiving in particular has been difficult to estimate. Prior approximations, however, indicate that over 16 billon hours of this type of care are given each year, representing a monetary value of more than $271.6 billion in 2020 (Alzheimer’s Association, 2022). Estimates of the cost of informal care have been established in previous research (Hurd et al., 2013; Kelley et al., 2015; Langa et al., 2001), but there have been no examinations of the relative differences in the monetary value of informal care by race and ethnicity.

## Racial and Ethnic Patterns in ADRD and Caregiving

Minoritized groups have less access to formal dementia care services (Lin et al., 2021). Black and Hispanic older adults who enter nursing homes with dementia typically do so at more advanced stages of disease progression than White older adults (Rivera-Hernandez et al., 2019). Moreover, Hispanic and Black older adults are more likely to be in racially segregated nursing homes with fewer resources and lower quality care than White older adults (Rivera-Hernandez et al., 2019). Notably, formal support utilization is associated with higher depressive symptoms for Hispanic caregivers (Rote et al., 2019). According to Rote et al. (2019), it is unclear if this association is due to aging-in-place preferences or if formal support helps Hispanic caregivers sustain their caregiving activities despite mental health challenges. The use of dementia care services has important implications for family members’ ability to sustain their caregiving.

The experience of informal caregiving varies across racial and ethnic groups, with non-White caregivers more likely to provide high-intensity informal care than White caregivers (Ali et al., 2022; Alzheimer’s Association, 2021). Intensity of caregiving is negatively related to self-rated health for White and Black caregivers but not Hispanic caregivers (Rote et al., 2019). However, Hispanic caregivers face additional challenges due to care recipients’ higher prevalence of chronic conditions, early-age onset of disability, and longer life expectancy with both (Aranda & Knight, 1997; Cantu et al., 2013). Black and Hispanic adults are both more likely to be informal caregivers than White adults (Trivedi et al., 2014), and Hispanic older adults are more likely to receive informal care through family networks than White older adults (Angel et al., 2014). Yet, while a great deal is known about the racial and ethnic differences in the use of informal care, little is known about racial and ethnic differences in the monetary value of informal care. Measures of differences in the monetary value of informal care between racial and ethnic groups help concretize the distributional consequences of disparities in dementia by illuminating the financial realities of different racial and ethnic groups. Importantly, concretizing the monetary cost of care experienced by minoritized racial and ethnic groups makes visible the potential need for intervention for all segments of the U.S. population through accessible supports (Aranda et al., 2021). Therefore, an investigation of monetary value has greater implications for health equity than a study of total hours of informal care.

## Costs of ADRD Caregiving

Caregivers for PwD incur high financial and non-financial costs. Previous efforts to calculate dementia care-related costs valued informal care as ranging on average between $41,689 and $56,290 annually per person (measured in 2010), suggesting that the total monetary cost of dementia was between $157 billion and $215 billion (Hurd et al., 2013). Informal care provision is often (though not exclusively) provided by family members (Aranda et al., 2021). But there is substantial heterogeneity in intergenerational family structures among racial and ethnic groups (Katz et al., 2020), which may influence the types and levels of informal care delivered and the costs of such care. One study found racial and ethnic differences in the availability of spouses and children to serve as potential caregivers, with White PwD being more likely to have a spouse caregiver than Black and Hispanic PwD (Choi et al., 2021). Conversely, Black and Hispanic PwD had more children available as potential caregivers than White PwD. While Choi et al. (2021) showed general differences in the availability of informal caregivers by race and ethnicity, they did not explicitly examine how these differences translated into racial and ethnic differences in the total hours of informal care or the monetary value of such care.

Recent work has documented racial and ethnic differences in the amount of informal caregiving for older adults. Rote et al. (2018) used time to show that Black and Hispanic caregivers engage in care activities more frequently than White caregivers. Another study using Health and Retirement Study (HRS) data showed that Hispanic and Black respondents with dementia had significantly higher odds of receiving *intensive informal care*, defined as ≥200 hours a month (Friedman et al., 2015). The authors concluded that racial and ethnic differences in the intensity of caregiving might be due to differences in proximity of informal caregivers or racial and ethnic differences in accessibility of formal care. These studies did not calculate costs and did not parse the total hours of informal care into component sources, such as care from spouses, children, and other family members and friends.

## Dementia in Minoritized Populations

Alzheimer’s disease is the most common type of dementia among older adults and typically has a lengthy duration of illness, with cognition and daily functioning declining over mild, moderate, and severe stages prior to death (Brück et al., 2021). Accurately assessing ADRD in large secondary data sets is an ongoing challenge. Notably, there is evidence to suggest that sensitivity (ability to correctly identify those with dementia) is typically higher among Black and Hispanic older adults than White older adults when using existing algorithms to identify ADRD prevalence, but specificity (ability to correctly identify those without dementia) is typically lower (Gianattasio et al., 2019). As a result, disparities between White older adults and racial and ethnic minoritized groups have been overestimated. This paper advances previous studies of racial disparities of dementia prevalence (Lin et al., 2021; Manly et al., 2022; Moon et al., 2019, 2023; Rajan et al., 2021) by applying a newly created algorithm (Gianattasio et al., 2020) to more accurately identify ADRD among non-Hispanic Blacks (henceforth Black), Hispanic, and White older adults and estimate the monetary value of informal caregiving to PwD among these racial and ethnic groups. Accurate estimates of prevalence of ADRD by racial and ethnic group not only present disparities more explicitly but also contribute to more precise cost estimation of ADRD caregiving.

## Conceptual Model of Racial and Ethnic Differences in Sources and Hours of Informal Care

Our objective is to estimate the average number of hours of informal care received by community-dwelling PwD across racial and ethnic groups and use these estimates to calculate the monetary value of informal care of PwD. We advance prior population-based studies on the value of informal care by using a dementia identification algorithm specifically designed for racial and ethnic comparisons. Our study uses data from the HRS, a nationally representative survey, to expand on Hurd et al.’s (2013) informal care value estimates in several ways. First, we use the *modified Hurd algorithm* (Gianattasio et al., 2020) to improve sensitivity and reduce bias by race and ethnicity to identify specific dementia cutoffs among Black, Hispanic, and White populations. Second, we stratify estimates by respondent race or ethnicity to examine differences in the value of informal care.

**Figure 1.**
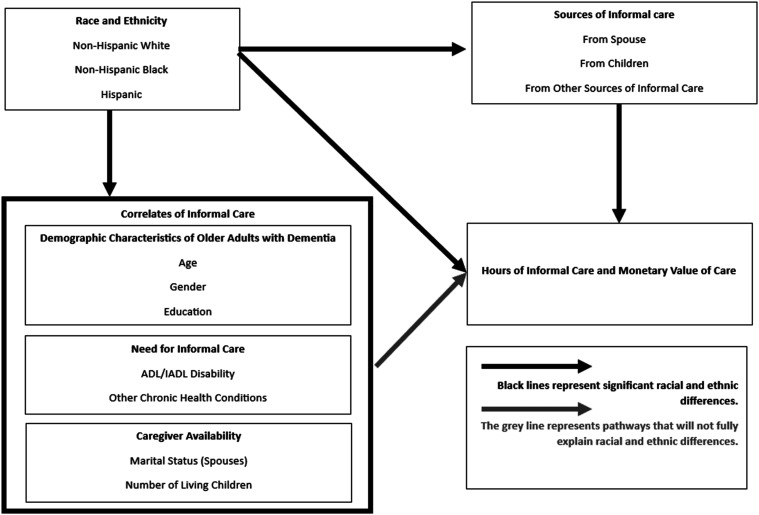
Conceptual model of racial and ethnic differences in sources and hours of informal care.

Figure 1 shows our conceptual model for understanding racial and ethnic differences in sources and hours of informal care. The lines from the race and ethnicity box to sources of informal care and hours of informal care boxes represent the hypothesized differences by racial and ethnic group. The line across the top shows that we expect that the source of care (spouse, child, or other) will vary by race and ethnicity (Choi et al., 2021). On the lower right-hand side of the model, we hypothesize we will find significant differences in the total hours of informal care by racial and ethnic group, with Black and Hispanic PwD receiving more hours of informal care (Ali et al., 2022; Kelley et al., 2015).

The box on the bottom left of the model shows potential correlates of informal care that may explain some of the racial and ethnic differences in hours of care, including demographics, need for informal care, and caregiver availability. *Need for care* includes disability in activities of daily living (ADL), instrumental activities of daily living (IADL), and other chronic health conditions. Black and Hispanic older adults have higher levels of disability and chronic health conditions than White older adults (Kelley-Moore & Ferraro, 2004; Mendes de Leon et al., 2005). Hispanic older adults also have longer life expectancy with functional limitation and chronic conditions (Aranda et al., 2021; Cantu et al., 2013) and are more likely to continue to live in community settings, even at more advanced stages of dementia, than White older adults (Thomeer et al., 2015), possibly necessitating higher levels of informal care. Caregiver availability that likely differs by race and ethnicity and therefore may explain group differences in hours of informal care is also included in correlates of informal care. Previous work on family availability found that being married and having at least one adult child is related to receiving more hours of informal care (Choi et al., 2021). Caregivers of Hispanic and Black older adults are less likely to be spouses and more likely to be other family members (Janevic & Connell, 2001). The larger sizes of Hispanic families may be associated with greater availability of informal care for Hispanic PwD than White and Black PwD. Conversely, lower probability of having a spouse in late life may decrease the hours of spousal care for Black PwD. The light gray arrow from correlates of informal care to hours of informal care represents an expectation that these factors will be significantly associated with hours of informal care but will not completely explain racial and ethnic differences in hours of informal care.

## Study Goals

By estimating total monetary value of informal caregiving for PwD by race and ethnicity in a nationally representative sample, we provide useful data to inform health equity efforts and the development of policy to address the care of PwD, tailored to the diverse and different informal care experiences across race and ethnicity. We aim to answer the following questions:

1) How do sources of informal care for PwD vary by racial and ethnic group?
2) How does the monetary value of informal care vary across racial and ethnic groups?

Prior research indicates that there will be significant racial and ethnic differences in both source of informal care as well as total hours of informal care. Furthermore, the racial and ethnic differences in total hours of informal care will be reflected in differences in the monetary value of informal care. It is worth noting that we use “value” to signify the market value of care and not the intrinsic value of care for care recipients or caregivers. The intrinsic value of informal care, as in appreciation felt by care recipients, might be the same across racial and ethnic groups, but the total monetary costs based on the same market value ($24) may be different because of differences in the amount of care.

## Method

### Data and Sample

We used the Health and Retirement Study (HRS) data from 2002 to 2018. The HRS is a longitudinal study that surveys people aged at least 51 years every two years to collect nationally representative data in the United States. Our sample included community-dwelling older adults over age 70 with dementia, as defined by the modified Hurd algorithm (Gianattasio et al., 2020), and at least one limitation of activity of daily living (ADL) or instrumental activity of daily living (IADL) (*n* = 10,015 person-observations). Dementia status was determined using the user-contributed Gianattasio-Power Predicted Dementia Probability Scores and Dementia Classifications pre-coded dementia diagnosis data on the HRS website. Respondents were categorized into three groups: non-Hispanic White, non-Hispanic Black, and Hispanic, based on self-reported race and ethnicity (Gianattasio et al., 2019). The modified Hurd algorithm only provides dementia categories for White, Black, and Hispanic older adults, so we were unable to include respondents from *other* racial or ethnic groups. In all, we included nine waves of the HRS, and study respondents (henceforth respondents) could contribute up to eight observations each.

### Measures

#### Caregivers

Caregiving in the HRS is measured in the context of help with ADL and IADL disability. Older adults who have ADL/IADL impairment are asked for details about who is helping with their disability. Older adults with ADL/IADL disabilities, or the person responding to the survey for them, reported the relationship between the PwD and helpers. Data about care provided by these helpers were used to identify all sources of care for each respondent in each wave. Information was also collected on how many days in a typical month, and how many hours on those days, they helped. Each older adult can have up to five named helpers.

We categorized caregivers into two broad groups: informal caregivers—including spouses, children (e.g., biological/adopted children, stepchildren, and children in-laws), and other family (e.g., siblings, parents, and grandchildren) and friends—and formal or professional caregivers who were paid for their services. Family and friend caregivers who reported being paid for their care were categorized as informal caregivers, regardless of the source of payment. Formal caregivers were categorized based on being listed as a paid professional or member of an organization.

#### Care Hours

We calculated *total hours of care per year* by multiplying the number of hours reported for each caregiver on a typical day by the reported numbers of days per year a caregiver typically provided care for IADL and ADL disability. Hours of care were top coded at 16 hours per day per caregiver, based on previous work (Friedman et al., 2015). Some respondents reported having multiple caregivers in a single category (such as multiple children who provided care), and hours of care in these categories were the sum of hours provided by each person in that category per day. To account for missing data, imputation of values was made empirically. Caregivers who reported 0 days of care in the past month were assumed to help one day every two months (*n* = 622 5% of all caregivers). Our assumption of one day of care every two months allowed us to keep infrequent caregivers, although their care could be less than once every two months. Caregivers who were missing days (*n* = 404 3% of all caregivers) or hours of care (*n* = 1418 12% of all caregivers) were imputed at the median hours of care for their category (spouse, child, other, or formal) for that survey wave due to right-skewed (high days and hours of care) distribution.

Due to excessive right skew (high) in number of days and hours for caregivers, we decided to use median hours for missing values to produce conservative estimates. We additionally examined our results using mean days and hours per type of caregiver per year as well as using a predictive model accounting for type of caregiver, race, age, gender, marital status, education, disability, and chronic conditions for each year. The results were substantively the same with slightly greater total hours of care when using the mean or predictive imputation assumptions. We provide additional information about imputed values of care in online supplementary materials. In our results, we present care hours per week for ease of interpretation.

#### Correlates of Informal Care

We examined whether racial and ethnic differences in hours of informal care were associated with respondents’ demographic characteristics (age and gender), education level (indicator of high school and above education), caregiver availability (number of living children: three categories of 0, 1–3, and 4 or more children; marital status: married/partnered or not), and respondents’ health (number of medical comorbidities, ranging 0–8; number of ADL/IADL limitations, ranging 0–11). We included eight medical comorbidities: hypertension, diabetes, cancer, chronic lung disease, stroke, heart problems, psychiatric problems, and arthritis. The 11 ADL/IADL domains assessed were dressing, walking across a room, bathing, eating, getting in/out of bed, using the toilet, preparing hot meals, shopping for groceries, making phone calls, taking medications, and managing money.

The inclusion of covariates in our regression analysis allowed us to examine if racial and ethnic differences in hours of informal care are robust to other correlates of care. Our conceptual model reflects a direct relationship between race and ethnicity of the PwD and total care hours. In our analyses, we first estimated the relationship between race and ethnicity and care hours and then examined the extent to which the racial and ethnic differences in hours of informal care are explained by differences in covariates, such as demographic characteristics, need for informal care, or potential sources of informal care.

### Statistical Analysis

**Figure 2.**
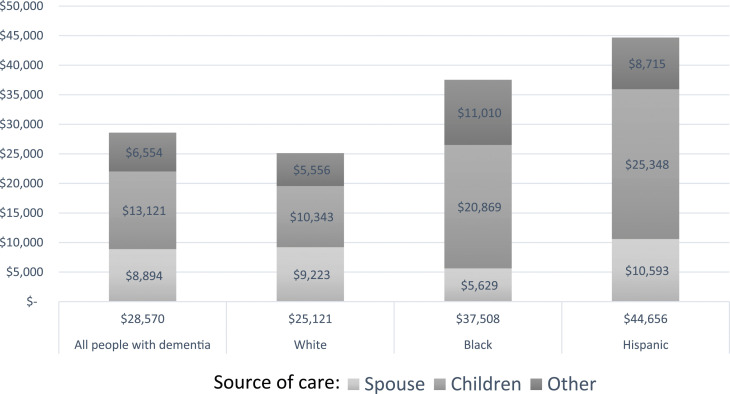
Average annual value of informal care for older adults with dementia (2018 U. S. Dollars).

We first described the composition of PwD in our sample by race and ethnicity (Table 1). We presented the proportion of older adults in each racial and ethnic group who reported each type of caregiver to show differences in the source of care by racial and ethnic group (Table 2). We then calculated the average weekly hours of care respondents reported receiving from each source by race and ethnicity (Table 3). We converted hours of care into total annual cost of care in 2018 using an average replacement cost of $24 an hour (John Hancock Life Insurance Company, 2018), consistent with previous research approaches (Hurd et al., 2013; Kelley et al., 2015) (Figure 2). Next, we conducted regression analysis to examine racial and ethnic differences in amount of care received (Table 4). Due to the high proportion of respondents who did not receive any assistance, we used a two-part model, with a logit model in the first part and a generalized linear model (GLM) with the log link and gamma distribution for the second part. The logit modeled the probability of receiving informal care, while the GLM modeled the hours of informal care conditional on receiving informal care. We used two sets of variables to model hours of informal care: Model 1 uses only race and ethnicity, and Model 2 predicts hours of informal care for each racial and ethnic group, controlling for demographics (age and gender), family (marital status and number of children), education, and health (number of medical comorbidities and number of ADL/IADL limitations). All analyses were conducted using STATA 17.0.

**Table 1.** Characteristics of Older Adults With Dementia, 2002–2018, Health and Retirement Study (*n* = 10,015).

	Total (*n* = 10,015)	By Race/Ethnicity						
		White (*n* = 7015 70%)	*p*-Value	Black (*n* = 1866 19%)	*p*-Value	Hispanic (*n* = 1134 11%)	*p*-Value	Three-Group Comparison *p*-Value
Age (years) (M, SD)	84.52 (6.39)	84.84 (5.94)	(Ref)	83.19 (8.22)	<.001	83.66 (7.20)	.000	<0.001
Female (%, *N*)	0.62 (6130)	0.60 (4133)	(Ref)	0.68 (1256)	.002	0.66 (741)	.021	0.004
Married (%, *N*)	0.39 (3959)	0.42 (3039)	(Ref)	0.24 (463)	<.001	0.37 (457)	.205	<0.001
Education (years) (M, SD)	10.86 (3.87)	11.78 (2.98)	(Ref)	9.13 (4.77)	<.001	5.77 (4.77)	<.001	<0.001
Living children (M, SD)	3.25 (2.29)	2.95 (1.87)	(Ref)	4.05 (3.54)	<.001	4.67 (3.19)	<.001	<0.001
ADL/IADL (0–11) (M, SD)	3.00 (3.31)	2.75 (3.01)	(Ref)	3.47 (4.25)	<.001	4.37 (4.05)	<.001	<0.001
Chronic conditions (0–8) (M, SD)	2.90 (1.52)	2.87 (1.44)	(Ref)	3.04 (1.86)	.005	2.90 (1.69)	.666	0.015

**Table 2.** Proportion of Older Adults With Dementia Receiving Care From Different Sources, 2002–2018, Health and Retirement Study, (*n* = 10,015).

	Total (*n* = 10,015)	By Race/Ethnicity						
		White (*n* = 7015 70%)	*p*-Value	Black (*n* = 1866 19%)	*p*-Value	Hispanic (*n* = 1134 11%)	*p*-Value	Three-Group Comparison *p*-Value
Any care	0.57	0.55	(Ref)	0.61**	.009	0.70***	<.001	<0.001
Any informal care	0.56	0.53	(Ref)	0.60**	.003	0.68***	<.001	<0.001
Spouse	0.18	0.20	(Ref)	0.11***	<.001	0.17	.219	<0.001
Child	0.36	0.33	(Ref)	0.43***	<.001	0.50***	<.001	<0.001
Other	0.18	0.16	(Ref)	0.29***	<.001	0.23***	<.001	<0.001
Any formal care	0.12	0.11	(Ref)	0.13	.282	0.16***	<.001	0.004

*p <= .05; **p <= .01; ***p <= .001.

**Table 3.** Average Weekly Hours of Care From Different Sources for Older Adults With Dementia, 2002–2018, Health and Retirement Study (Mean and Standard Deviation) (*n* = 10,015) (Unadjusted).

	Total (*n* = 10,015)	By Race/Ethnicity						
		White (*n* = 7015 70%)	*p*-Value	Black (*n* = 1866 19%)	*p*-Value	Hispanic (*n* = 1134 11%)	*p*-Value	Three-Group Comparison *p*-Value
Total hours of care (M, SD)	26.5 (45.0)	23.5 (40.4)	(Ref)	33.5 (61.4)	<.001	41.0 (56.1)	<.001	<0.001
Hours of informal care (M, SD)	22.9 (41.1)	20.1 (36.6)	(Ref)	30.1 (57.9)	<.001	35.8 (52.3)	<.001	<0.001
Spouse (M, SD)	7.1 (22.4)	7.4 (21.4)	(Ref)	4.5 (21.8)	<.001	8.5 (27.9)	.440	0.002
Child (M, SD)	10.5 (27.5)	8.3 (23.2)	(Ref)	16.7 (40.1)	<.001	20.3 (41.2)	<.001	<0.001
Other (M, SD)	5.3 (21.5)	4.5 (19.2)	(Ref)	8.8 (34.1)	<0.001	7.0 (21.8)	<.001	<0.001
Hours of formal care (M, SD)	3.6 (16.6)	3.4 (15.9)	(Ref)	3.4 (17.5)	.960	5.2 (19.6)	.027	0.035

**Table 4.** Two-Part Logistic and Gaussian Model Predicting Hours of Informal Care for Older Adults With Dementia, 2002–2018, Health and Retirement Study (*n* = 10,015).

	Race/Ethnicity Model 1	Adjusted Model 2
	*b* (95 ci)	*b* (95 ci)
Logit model predicting receiving informal care		
Race/ethnicity (ref: NH White)		
NH Black	1.33** (1.11, 1.34)	1.59 (1.07, 0.85)
Hispanic	1.88*** (1.60, 1.12)	2.22 (0.87, 0.67)
Age		1.01 (1.00, 1.03)
Female		1.29** (1.07, 1.57)
Married		1.71*** (1.41, 2.06)
Number of living children (ref: None)		
1–3		1.84** (1.28, 2.64)
4+		1.90** (1.30, 2.78)
HS education		0.88 (0.76, 1.01)
Number of ADL/IADL		3.64*** (3.13, 4.22)
Number of chronic conditions		1.07** (1.02, 1.12)
Gaussian model predicting hours of informal care dependent on receiving care		
Race/ethnicity (ref: NH White)	1.30*** (1.18, 1.37)	1.25*** (1.15, 1.37)
NH Black	1.38*** (1.24, 1.37)	1.24*** (1.13, 1.37)
Hispanic		
Age		1.00 (1.00, 1.00)
Female		1.07 (0.97, 1.18)
Married		1.39*** (1.26, 1.54)
Number of living children (ref: None)		
1–3		1.25** (1.08, 1.45)
4+		1.34*** (1.13, 1.59)
HS education		0.94 (0.88, 1.00)
Number of ADL/IADL		1.19*** (1.17, 1.20)
Number of chronic conditions		1.02 (0.99, 1.05)
*n*	10,015	10,015

*p <= .05; **p <= .01;***p <= .001.

We accounted for the HRS complex survey design using STATA’s svy command. We used sampling weights that reflect the estimates of the U.S. population over age 70 in a given year, so respondents with multiple years of data had different sampling weights specific to the survey year. As a sensitivity test, we also conducted our analysis using the *expert model*, which may offer advantages when making comparisons across racial and ethnic groups (Gianattasio et al., 2020). The expert model is a logistic model that predicts dementia status for self-respondents and proxy interviewees. The model covariates include sociodemographic, cognitive and physical health, and social engagement characteristics, all of which were chosen based on the expertise of an ADRD researcher and the epidemiologic evidence (Gianattasio et al., 2020). Results from our group comparisons, using the expert algorithm, were substantively the same as when using the modified Hurd algorithm (results available upon request). Of note, the Hispanic sample identified using the expert model was smaller, had worse health, and received more hours of informal care, suggesting that this algorithm identified only the most impaired Hispanics with dementia.

## Results

Table 1 presents the characteristics of PwD identified using the modified Hurd algorithm. Most of the sample was White (70%), followed by Black (19%) and Hispanic (11%). There were significant group differences in demographics, caregiver availability, and health characteristics of PwD. Black (83.19 years) and Hispanic (83.66 years) PwD were significantly younger than White PwD (84.84 years). Black (68%) and Hispanic (66%) PwD were more likely to be female than White PwD (60%). White PwD had significantly more years of education (11.78 years) than Black (9.13 years) and Hispanic (5.77 years) PwD. Caregiver availability differed by race and ethnicity: fewer Black PwD were married (24%) than White PwD (42%), and Black (4.05) and Hispanic (4.67) PwD had significantly more living children than White PwD (2.95). Health of PwD was generally worse for minorities: Black (3.47) and Hispanic (4.37) PwD had more ADL and IADL than White PwD (2.75), and Black PwD had significantly more chronic conditions (3.04) than White PwD (2.90).

Table 2 shows the proportion of PwD receiving care from different sources. Slightly more than half of all PwD were receiving some assistance (57%). Slightly more than half of all PwD also received some form of informal care (56%). Among the total sample of PwD, 33% received care from at least one child, 20% from a spouse, and 16% from other sources of informal care. Significantly higher proportions of Hispanic PwD than White PwD received care from all sources examined, except informal spousal care. A significantly higher portion of White PwD (20%) received informal care from a spouse than Black PwD (11%). Conversely, White PwD (33%) were less likely to receive informal care from children than Black (43%) and Hispanic (50%) PwD. A significantly higher proportion of Black PwD (29%) received informal care from friends and family other than children and spouses than White (16%) and Hispanic (23%) PwD. Proportions of PwD receiving formal paid care were similar for White (11%) and Black (13%) PwD, while a significantly higher proportion of Hispanic PwD (16%) received paid care.

Table 3 shows the weekly hours of care from each source of care by race and ethnicity. On average, PwD received 22.9 hours of informal care and 3.6 hours of formal care per week, with 26.5 average total care hours per week. White PwD received significantly fewer hours of informal care (20.1 hours) in an average week than Black (30.1 hours) and Hispanic (35.8 hours) PwD. Sources of informal care varied across groups, with spouses providing more hours of care for White PwD (7.4 hours) than Black PwD (4.5 hours). Hispanic PwD received the most hours of care from children (20.3 hours), followed by Black (16.7 hours) and White (7.4 hours) PwD. Black PwD (8.8 hours) received more hours of care from *other* (non-spouse and non-child) informal caregivers than Hispanic (7.0 hours) and White (4.5 hours) PwD.

Figure 2 converts the average hours of weekly care from Table 3 into the annual monetary value (in 2018 USD) of informal care. In our sample, the overall average annual monetary value of informal care for PwD was $28,570 a year, with substantial racial and ethnic differences. The total monetary value of informal care by race and ethnicity was $25,121 for White PwD, $37,508 for Black PwD, and $44,646 for Hispanic PwD. The monetary value of spousal care was greater for White ($9223) and Hispanic ($10,593) PwD than for Black PwD ($5629). Conversely, the monetary value of care from children was substantially higher for Hispanic ($25,348) and Black ($20,869) PwD than for White PwD ($10,343). Other sources of informal care had more monetary value for Black PwD ($11,010) than for Hispanic ($8715) and White ($5556) PwD.

Table 4 shows the results of the two-part models predicting total hours of informal care. Model 1 shows the odds of receiving any informal care in the upper panel and the comparative hours of care depending on receiving care in the lower panel. Model 1, without covariates, shows that Hispanic and Black PwD were significantly more likely to receive informal care; the lower panel shows that Hispanic and Black PwD also received more hours of informal care than White PwD.

Model 2 adds control variables for factors related to care needs. The top panel shows there is no significant racial and ethnic difference in the odds of receiving any care after controlling for demographics, family structure, education, and health of the PwD. The bottom panel shows that among PwD receiving care, significant racial and ethnic differences in *hours* of informal care received held even with the inclusion of covariates. Black and Hispanic PwD still received significantly more hours of informal care than White PwD when controlling for covariates.

## Discussion

The current study used nationally representative survey data from the HRS and a novel algorithmic approach to identifying dementia cases in diverse populations to calculate the monetary value of informal caregiving provided to community-dwelling PwD. We extended previous research on costs of informal care (Hurd et al., 2013) by examining racial and ethnic differences in sources of informal care and used an updated algorithm for identifying racial and ethnic differences in dementia prevalence more accurately.

Consistent with previous work, our results demonstrate the significant cost of care for PwD provided by unpaid family and friends. Importantly, we show that there are racial and ethnic differences in informal caregiving for PwD. We extend previous research (Choi et al., 2021) by showing how the racial and ethnic differences in total hours of informal care for White PwD and Black PwD are partially due to family structure and caregiver availability (e.g., White PwD being more likely to be married). White PwD are more likely than Black PwD to receive care from a spouse, who in turn delivered more hours of spousal care. Likewise, similar to other studies, we found that children were larger sources of care for Hispanic and Black PwD than for White PwD. While children were the largest source of care for all racial and ethnic groups, they provided significantly more hours of care for Hispanic and Black PwD, providing twice as many hours on average for Black PwD compared to White PwD. For Hispanic PwD, children provided an average of 20 hours of care per week. Previous research has shown the importance of other non-spouse and non-child caregivers for minoritized groups (Ali et al., 2022; Mehta & Yeo, 2018; Silverstein & Litwak, 1993). Our study is the first to our knowledge to compare estimates of the number of hours and monetary value that these other care sources deliver.

We were unable to calculate the forgone wages of caregivers in our estimates of racial and ethnic differences in the monetary value of informal caregiving. Prior research used models that set the value of forgone wages of informal caregivers as less than the cost of replacement care (Hurd et al., 2013). Informal caregivers of minoritized PwD may have lower market wages than White caregivers due to lower levels of education, discrimination, or both (Lang & Spitzer, 2020) and therefore earn lower foregone wages. Opportunity costs of informal care to caregivers include forgone wages for the actual hours worked as well as forgone education and career development, which are not considered when using forgone wages as the cost of care. Differences in the opportunity costs using forgone wages may appear to decrease the racial and ethnic inequality in the monetary value of informal care for PwD.

It is clear that the intergenerational nature of informal caregiving for Black and Hispanic PwD relative to White PwD represents major differences in forgone employment and education, highlighting potentially critical differences in opportunity costs related to caregiving (Chari et al., 2015). The monetary costs of care appear to weigh heavily on children in racial and ethnic minoritized groups. Such costs likely influence life course trajectories among racial and ethnic minoritized groups, including workforce participation and their own health and well-being (Aranda et al., 2021). For example, caregiving responsibilities make workforce participation less likely, increase role strain, and lead to higher levels of depressive symptoms (Martsolf et al., 2020; Wang et al., 2011; Wilson et al., 2007).

Previous research has documented the unique challenges faced by children and grandchildren caring for older family members, including negative impacts on financial resources, educational trajectories, and personal health and well-being (Chirico et al., 2021; Steiner & Fletcher, 2017). The differences in hours of informal care by children for Hispanic and Black PwD relative to White PwD represent important differences in opportunities to improve wages (e.g., accepting a new job or promotion) or gain additional training, which in turn magnify the overall opportunity cost of caregiving for these minoritized populations. Our findings imply that the impact of caregiving duties on lost opportunity for advancement is not the same across racial and ethnic groups. Such differences can contribute to established disparities in wealth building, home ownership, and attainment of higher degrees (Pfeffer & Schoeni, 2016).

While the current research shows that patterns in informal caregiving may lead to negative effects on racial and ethnic disparities in financial outcomes, it should be acknowledged that caregiving may also result in positive outcomes, including satisfaction, meaning, and reward (see Quinn & Toms, 2019 for a review). For Hispanic caregivers, positive appraisal of caregiving experiences is related to lower levels of depression and greater life satisfaction (Morano, 2003). Black caregivers may derive higher levels of spiritual meaning from caregiving than White caregivers (Farran et al., 1997; Robinson-Lane & Zhang, 2018). More research on the experience of dementia caregiving across racial and ethnic groups is needed to better understand the qualitative costs as well as the strengths and rewards among diverse family members above and beyond monetary assessments.

While prior cost estimates have made efforts to portion out the amount of care directly attributable to dementia (Daras et al., 2017; Kelley et al., 2015; Langa et al., 2001), our focus is to document racial and ethnic differences in the care of PwD more broadly. We estimated the total hours of care that PwD received from informal sources, regardless of the primary reason for care. This approach more closely represents the experiences of caregivers of PwD who do not parse out the portion of their care based on the cause of the need for care. Nevertheless, our estimates align well with an earlier study that estimated the final five-year cost of informal care for PwD who died to be $83,022, when using a $20 imputed hourly wage value in 2010 USD (Kelley et al., 2015). This is roughly 30% lower than the costs we estimated using a $24 imputed wage in 2018 USD ($28,569 per year for five years, $142,848). Our sample is not necessarily directly comparable to previous studies, and our estimates appear to be higher than previous studies.

Finally, our multivariate analyses show that for PwD receiving any assistance, racial and ethnic differences in the intensity of care are not fully explained by differences in family structure and potential caregiver availability, functional impairment, or physical health. Our results suggest that differences in utilization of informal care may come from other sources of racial and ethnic variation, including lack of information (Napoles et al., 2010) and cultural norms, such as familialism or beliefs about dementia (Abdulrahim et al., 2023; Sayegh & Knight, 2013). Additionally, we have not examined the role of family finance, which plays an important role in determining what types of formal care alternatives are available (Aranda et al., 2021).

## Limitations and Future Directions

Though the findings reported here offer unique insights into the costs of ADRD across racial and ethnic groups, several limitations should be recognized. Estimates in Table 3 represent average hours per week from each source of care among all people with dementia; averages include zeros for PwD who do not receive care from these sources. As a result, the estimates of actual care received on average from those receiving care from each source would be higher. Therefore, our estimates do not necessarily represent the high burden of care on caregivers, but rather they are estimates of the total number of hours of care in the population. Caregivers of people with dementia would likely provide more hours of care than what our average estimates. Our sample represents a cohort of PwD between 2002 and 2018 and does not reflect the population with dementia in any given year. Our sample selection is a cross section from a longitudinal study in which individuals can contribute multiple person years, and our findings are limited to descriptions of the HRS population. Future research should move beyond our descriptive models into predictive models of informal care. Our results did not substantively change when using any single wave of the HRS, but the standard errors were larger due to smaller sample sizes. Of note, we could not capture the evolving impacts related to the COVID-19 pandemic, which resulted in increased caregiving time and burden for some families (Hwang et al., 2021) and may have differentially impacted non-White caregivers (Moon et al., 2022). Racial and ethnic disparities in informal care intensity may change in the post-pandemic period, and continued research in this area will be critical.

Our monetary value estimates are expressed in 2018 USD and serve our study goal of describing the total number of hours of informal care as reported in the HRS by race and ethnicity. Future work should undertake a more comprehensive analysis of the monetary value of informal care, incorporating additional validated metrics, for example, regional differences in cost of replacement care for dementia.

Additionally, our analysis is limited to examining racial and ethnic differences among White, Black, and Hispanic PwD. Our exclusion of other racial and ethnic group limits the comprehensiveness of our study for the broader U.S. population. Future work should incorporate algorithms to identify dementia in Asian, Native American, Arab/Middle Eastern/North African, and multiracial populations.

It is likely that caregiving responsibilities captured in our estimates of child informal care are shared by multiple children. While outside the scope of this study, the use of multiple caregivers complicates the potential economic burden of caregiving for caregivers. Averaging the potential costs of caregiving across multiple caregivers obscures the potential for identifying high burdens of care for some caregivers and less burden for others. For example, while multiple caregivers are common among Hispanic households, primary caregivers nevertheless may vary substantially in their caregiving role compared to others. Some caregivers are adult children who live with their parents and have no family of their own, while others move their older adult family members into their own household (Cantu & Angel, 2017). Future work on racial and ethnic differences in forgone wages and economic burden on caregivers will need to examine the role of family structure and presence of multiple caregivers in informal care.

Taken together, the current findings underline the impact of informal caregiving on communities and society as a whole and highlight the need for interventions and policy to be tailored to the unique needs of a growing aging population in the United States, which is increasing in racial and ethnic diversity (Dilworth-Anderson et al., 2020). ADRD has profound negative impacts on individuals, families, and society, but these impacts are not uniform across populations. Our findings add to previous caregiving research that demonstrates crucial differences in how families care for PwD. In line with the RAISE Act and the 2022 National Strategy to Support Family Caregivers (Administration for Community Living, 2022), the needs of caregivers from historically marginalized racial and ethnic groups must be attended to through community-based public health initiatives, economic policies to support families and older adults, and interventions to reduce barriers to services.

## Supplemental Material

Supplemental Material - Racial and Ethnic Disparities in the Monetary Value of Informal Caregiving for Non-Institutionalized People Living With Dementia Supplemental Material for Racial and Ethnic Disparities in the Monetary Value of Informal Caregiving for Non-Institutionalized People Living With Dementia by Philip Cantu, Tsai-Chin Cho, Mary Wyman, Brooke Helppie-McFall, and Kristine Ajrouch in Journal of Aging and Health.
