# Supplementary material for: Racial and Ethnic Disparities in the Monetary Value of Informal Caregiving for Non-Institutionalized People Living With Dementia
Source: J Aging Health. 2024 Jun 17;36(9):570–82. doi: 10.1177/08982643241262917 (PMC11363470; doi:10.1177/08982643241262917)
Supplement: Supplemental Material - Racial and Ethnic Disparities in the Monetary Value of Informal Caregiving for Non-Institutionalized People Living With Dementia [file sj-pdf-1-jah-10.1177_08982643241262917.pdf]

Online Methods Supplement

Methods Supplement 1: Sample with dementia identified by the modified Hurd algorithm, by survey year, HRS 2002-2018
Methods Supplement 2: Distribution of types of caregivers per year and missing information on days and hours, HRS 2002-2018
Method Supplement 3: Comparison of missing value replacement for median, mean, and predictive imputation, by year and type of caregiver, HRS 2002-2018
Methods Supplement 4: Comparison of average hours of care by method of imputation

For transparency in our imputation of missing data, we provide the following supplemental materials.
Supplement 1 describes the total number of caregivers with less than 1 day of care a month (imputed as 6 days a year), those missing number of days of care and those who we truncated their care days at 16 hours per day for each year of the HRS.
Supplement 2 describes the distribution of missing care days and hours by type of caregiver for each year of the HRS.
Supplement 3 describes the replacement values when using median or mean values for type of caregivers in a given year as well as the average replacement value when imputing using a predictive model for missing hours and days accounting for type of caregiver, race, age, gender, marital status, education, disability, and chronic conditions for each year
Supplement 4 shows the average hours of care by race under different imputation methods. All results are substantively the same under all three imputation models.

Methods Supplement 1: Sample with dementia identified by the modified Hurd algorithm, by survey year, HRS 2002-2018

Wave	Total with dementia	Community dwelling	Nursing home	n with caregivers	n caregivers	Less than 1 day care month	Missing Care days	Hours truncated at 16
2002	1498	1175	323	694	1277	55	30	113
2004	1493	1160	333	688	1306	63	49	136
2006	1463	1152	311	671	1312	55	28	114
2008	1474	1161	313	654	1222	52	24	137
2010	1564	1222	342	729	1558	73	51	120
2012	1616	1270	346	749	1614	81	41	131
2014	1503	1186	317	678	1470	96	61	99
2016	1376	1095	281	623	1359	78	65	115
2018	1093	899	194	523	1101	69	55	83
Total	13080	10320	2760	6009	12219	622	404	1048

Methods Supplement 2: Distribution of types of caregivers per year and missing information on days and hours, HRS 2002-2018

	Professional Caregivers			Spouse Caregivers			Child Caregivers			Other Caregivers			All Caregivers	
	n	n missing care days	n missing care hours	n	n missing care days	n missing care hours	n	n missing care days	n missing care hours	n	n missing care days	n missing care hours	n missing care days	n missing care hours
2002	132	4	6	201	3	22	625	9	58	319	14	36	30	122
2004	91	9	15	217	5	21	640	22	80	358	13	40	49	156
2006	103	2	8	215	2	23	632	16	66	362	8	36	28	133
2008	112	0	8	221	1	16	586	14	65	303	9	21	24	110
2010	105	5	13	238	7	23	752	23	91	463	16	50	51	177
2012	263	7	19	249	1	26	789	23	100	313	10	41	41	186
2014	227	8	16	217	5	26	749	30	118	277	18	49	61	209
2016	218	17	22	189	7	19	687	27	105	265	14	40	65	186
2018	156	10	14	175	6	16	550	20	77	220	19	32	55	139
Total	1407	62	121	1922	37	192	6010	184	760	2880	121	345	404	1418

Method Supplement 3: Comparison of missing value replacement for median, mean, and predictive imputation, by year and type of caregiver, HRS 2002-2018

	Paid Caregiver		Spouse Caregiver		Child Caregiver		Other Caregiver	
	Replacement value for missing days	Replacement value for missing hours	Replacement value for missing days	Replacement value for missing hours	Replacement value for missing days	Replacement value for missing hours	Replacement value for missing days	Replacement value for missing hours
	Median Replacement Values							
2002	235.5	3.0	353.2	4.0	333.0	3.0	188.4	3.0
2004	235.5	3.0	365.0	4.0	235.5	3.0	156.4	3.0
2006	260.7	3.0	365.0	4.0	235.5	2.0	156.4	3.0
2008	254.0	2.0	365.0	4.0	235.5	3.0	182.5	4.0
2010	235.5	3.0	365.0	3.0	117.7	3.0	141.3	3.0
2012	208.6	4.0	365.0	4.0	176.6	3.0	156.4	3.0
2014	208.6	5.0	365.0	4.0	141.3	3.0	141.3	3.0
2016	208.6	5.0	365.0	4.0	141.3	3.0	104.3	3.0
2018	176.6	5.0	365.0	3.0	141.3	3.0	82.4	3.0
	Mean Replacement values							
2002	212.2	4.5	292.3	6.7	216.2	4.5	201.7	5.0
2004	227.2	4.6	315.6	7.2	213.5	4.8	185.9	4.9
2006	241.4	5.1	325.5	6.5	211.3	4.5	189.0	4.8
2008	221.1	3.8	322.4	6.6	213.6	4.6	196.2	5.4
2010	229.3	4.2	304.4	5.5	180.7	4.3	175.2	4.9
2012	198.2	5.9	304.7	6.5	200.4	4.4	191.8	4.4
2014	196.0	5.9	312.4	6.4	186.4	4.3	173.4	4.3
2016	204.1	6.4	294.4	6.5	185.9	4.9	175.6	4.5
2018	191.0	6.2	308.7	5.4	185.5	5.0	145.3	4.3
	Predictive imputation							
2002	153.0	2.8	292.0	6.3	224.1	4.6	183.7	4.6
2004	203.0	3.7	301.0	6.3	204.0	4.6	195.1	4.8
2006	209.9	4.5	296.8	6.3	202.6	4.7	199.9	4.5
2008	N/A	3.9	353.4	6.9	202.9	4.3	214.2	5.2
2010	240.2	3.7	301.2	5.4	178.6	4.1	173.1	4.9
2012	177.1	5.3	304.2	6.6	190.3	4.5	196.0	4.2
2014	200.5	6.7	313.4	6.5	186.1	4.0	176.0	4.0
2016	198.5	5.6	279.9	6.4	161.1	4.7	157.3	3.9
2018	209.9	6.2	314.5	6.4	176.9	4.8	153.2	4.6

Methods Supplement 4: Comparison of average hours of care by method of imputation

	Total (n=10,015)	By race/ethnicity						Three group comparison p-value
		Non-Hispanic White (n=7,015 70%)	p-value	Non-Hispanic Black (n=1,866 19%)	p-value	Hispanic (n=1,134 11%)	p-value	
				Median				
Total Hours of Care (M, SD)	1,376 (2,339)	1,223 (2,102)	(ref)	1,741 (3,191)	<0.001	2,133 (2,915)	<0.001	<0.001
Hours Informal Care (M, SD)	1,190 (2,140)	1,047 (1,901)	(ref)	1,563 (3,010)	<0.001	1,861 (2,718)	<0.001	<0.001
Spouse (M, SD)	371 (1,162)	384 (1,112)	(ref)	235 (1,136)	<0.001	441 (1,451)	0.440	0.002
Child (M, SD)	547 (1,431)	431 (1,205)	(ref)	870 (2,085)	<0.001	1,056 (2,143)	<0.001	<0.001
Other (M, SD)	273 (1,116)	231 (1,000)	(ref)	459 (1,771)	<0.001	363 (1,134)	<0.001	<0.001
Hours Formal Care (M, SD)	185 (864)	176 (827)	(ref)	178 (912)	0.960	272 (1,017)	0.0269	0.035
				Mean				
Total Hours of Care (M, SD)	1,413 (2,361)	1,256 (2,124)	(ref)	1,793 (3,218)	<0.001	2,175 (2,925)	<0.001	<0.001
Hours Informal Care (M, SD)	1,224 (2,160)	1,077 (1,920)	(ref)	1,614 (3,036)	<0.001	1,900 (2,731)	<0.001	<0.001
Spouse (M, SD)	381 (1,175)	396 (1,125)	(ref)	243 (1,150)	<0.001	450 (1,460)	0.470	0.003
Child (M, SD)	562 (1,445)	444 (1,216)	(ref)	898 (2,110)	<0.001	1,078 (2,152)	<0.001	<0.001
Other (M, SD)	280 (1,128)	237 (1,011)	(ref)	473 (1,786)	<0.001	373 (1,147)	<0.001	<0.001
Hours Formal Care (M, SD)	189 (870)	180 (834)	(ref)	179 (916)	0.995	274 (1,020)	0.031	0.037
				Predictive Model				
Total Hours of Care (M, SD)	1,409 (2,359)	1,252 (2,122)	(ref)	1,789 (3,215)	<0.001	2,172 (2,925)	<0.001	<0.001
Hours Informal Care (M, SD)	1,221 (2,158)	1,074 (1,918)	(ref)	1,610 (3,034)	<0.001	1,898 (2,732)	<0.001	<0.001
Spouse (M, SD)	381 (1,175)	395 (1,124)	(ref)	243 (1,152)	<0.001	451 (1,460)	0.463	0.003
Child (M, SD)	561 (1,443)	442 (1,215)	(ref)	895 (2,107)	<0.001	1,075 (2,151)	<0.001	<0.001
Other (M, SD)	279 (1,127)	237 (1,010)	(ref)	471 (1,784)	<0.001	372 (1,146)	<0.001	<0.001
Hours Formal Care (M, SD)	187 (869)	178 (832)	(ref)	179 (915)	0.987	274 (1,020)	0.028	0.035
